# Supplementary material for: Experimental Investigation on the Effect of Graphene Oxide Additive on the Steady-State and Dynamic Shear Properties of PDMS-Based Magnetorheological Elastomer
Source: Polymers (Basel). 2021 May 28;13(11):1777. doi: 10.3390/polym13111777 (PMC8197886; doi:10.3390/polym13111777)
Supplement: Supplementary file 1 [file polymers-13-01777-s001.zip › polymers-1223518-supplementary.pdf]

polymers

MDPI

Article

Experimental Investigation on the Effect of Graphene Oxide Additive on the Steady-State and Dynamic Shear Properties of PDMS-Based Magnetorheological Elastomer

Minzi Liu, Mei Zhang *, Jiangtao Zhang *, Yanliang Qiao and Pengcheng Zhai

Hubei Key Laboratory of Theory and Application of Advanced Materials Mechanics, School of Science, Wuhan University of Technology, Wuhan 430070, China; liuminzi@whut.edu.cn (M.L.); QiaoYanliang@whut.edu.cn (Y.Q.); pczhai@126.com (P.Z.)

* Correspondence: zhangmei@whut.edu.cn (M.Z.); zhjiangtao@whut.edu.cn (J.Z.); Tel.: +86-27-8765-1820

Citation: Liu, M.; Zhang, M.; Zhang, J.; Qiao, Y.; Zhai, P. Experimental Investigation on the Effect of Graphene Oxide Additive on the Steady-State and Dynamic Shear Properties of PDMS-Based Magnetorheological Elastomer. *Polymers* **2021**, *13*, 1777. https://doi.org/10.3390/polym13111777

Academic Editor: Fang-Chyou Chiu and Kartik Behera

Received: 30 April 2021
Accepted: 26 May 2021
Published: 28 May 2021

Publisher's Note: MDPI stays neutral with regard to jurisdictional claims in published maps and institutional affiliations.

Copyright: © 2021 by the authors. Licensee MDPI, Basel, Switzerland. This article is an open access article distributed under the terms and conditions of the Creative Commons Attribution (CC BY) license (http://creativecommons.org/licenses/by/4.0/).

1. Viscosity of the GO/PDMS pre-polymer emulsion

The viscosity of the GO/PDMS pre-polymer emulsion after THF evaporated was tested by a rotational rheometer (MCR302, Anton Paar Company, Austria) with the shear rate varying from 0.01 to 100 s^{-1}. The viscosity curves of GO/PDMS emulsion are exhibited in Figure S1. It can be seen that the viscosity of the emulsion increases greatly with increasing GO content. This result is mainly due to the strong physical cross-linking between GO sheets and PDMS chains, which causes that one polymer chain can be adsorbed onto more than one GO sheet, thus forming the polymer bridging between GO fillers. As a result, a three-dimensional network is developed over the emulsion, which leads to high viscosity due to the restoring forces of extended bridges under the shear flow. It is worth noting that, for a shear rate lower than 0.1/s, a shear thickening phenomenon is present in the GO/PDMS pre-polymer emulsion, which does not occur in the pure PDMS pre-polymer. This is because of the nonlinear elasticity of polymer bridges and the shear-induced bridging occurring in the low shear rate range. That is, the shear flow induces more polymer chains adsorbed onto the GO sheets due to the strong physical cross-linking between the GO sheets and PDMS pre-polymer. Hence, the flow becomes shear-thickening [1,2].

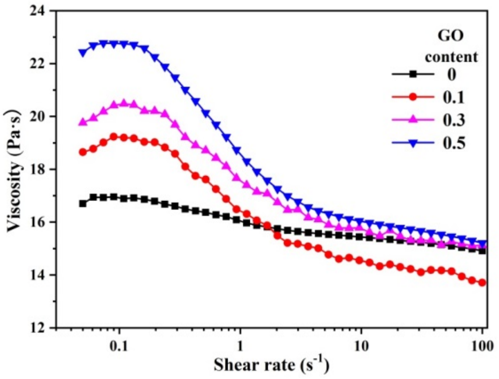

Figure S1. The viscosity curves of GO/PDMS pre-polymer emulsion.

2. Loss modulus of the GO-filled PDMS-based MREs

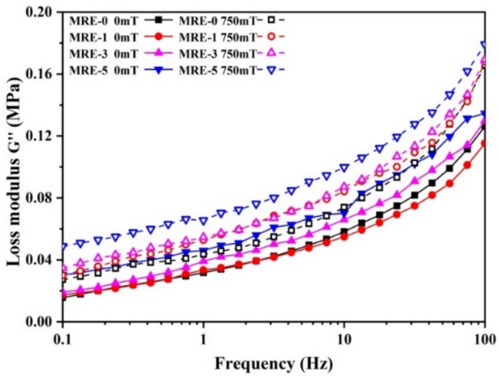

Figure S2. The loss modulus of the GO-filled PDMS-based MREs tested by the frequency sweeps under the zero-field and the magnetic saturation.

The effect of GO nanosheets on the loss modulus G″ of the MREs is plotted in Figure S2. It is apparent that, except for the MRE sample with 0.5 wt% GO sheets, which has an obviously higher G″, all other MREs show very small differences in G″. The increase of G″ in MRE with 0.5 wt% GO sheets is caused by the increased aggregations of GO and CIPs, which cause the high energy dissipation associated with the filler–filler interaction and the friction at filler–matrix interfaces [3]. In the MREs filled with 0.1 and 0.3 wt%, the increased energy dissipation associated with the friction at filler–matrix interfaces is compensated by the decreased internal damping of the PDMS matrix, since the chemical cross-linking of PDMS chains is inhibited by the GO sheets, and more linear structures of PDMS chains were produced in the MREs. Under the magnetic field, the values of G″ increase due to the increased interface energy dissipation between the fillers (GO and CIPs) and PDMS matrix and the increased internal damping of the PDMS matrix [4].

Supplementary References

1. Kamibayashi, M.; Ogura, H.; Otsubo, Y. Shear-thickening flow of nanoparticle suspensions flocculated by polymer bridging. *J. Colloid Interface Sci.* **2008**, *321*, 294–301.
2. Otsubo, Y.; Umeya, K. Rheological Properties of Silica Suspensions in Polyacrylamide Solutions. *J. Rheol.* **1984**, *28*, 95.
3. Poojary, U.R.; Gangadharan, K.V. Magnetic field and frequency dependent LVE limit characterization of magnetorheological elastomer. *J. Braz. Soc. Mech. Sci. Eng.* **2017**, *39*, 1365–1373.
4. Poojary, U.R.; Hegde, S.; Gangadharan, K.V. Dynamic blocked transfer stiffness method of characterizing the magnetic field and frequency dependent dynamic viscoelastic properties of MRE. *Korea-Aust. Rheol. J.* **2014**, *28*, 301–313.
